# Untangling the Contribution of the Subcomponents of Working Memory to Mathematical Proficiency as Measured by the National Tests: A Study among Swedish Third Graders

**DOI:** 10.3389/fpsyg.2016.01062

**Published:** 2016-07-18

**Authors:** Carola Wiklund-Hörnqvist, Bert Jonsson, Johan Korhonen, Hanna Eklöf, Mikaela Nyroos

**Affiliations:** ^1^Department of Psychology, Umeå UniversityUmeå, Sweden; ^2^Umeå Center for Functional Brain ImagingUmeå, Sweden; ^3^Faculty of Education and Welfare Studies, Åbo Akademi UniversityVaasa, Finland; ^4^Department of Applied Educational Science, Umeå UniversityUmeå, Sweden; ^5^Department of Education, Umeå UniversityUmeå, Sweden

**Keywords:** National tests, mathematics, working memory, pupils, education, cognitive profiles

## Abstract

The aim with the present study was to examine the relationship between the subcomponents in working memory (WM) and mathematical performance, as measured by the National tests in a sample of 597 Swedish third-grade pupils. In line with compelling evidence of other studies, individual differences in WM capacity significantly predicted mathematical performance. Dividing the sample into four groups, based on their mathematical performance, revealed that mathematical ability can be conceptualized in terms of different WM profiles. Pupils categorized as High-math performers particularly differed from the other three groups in having a significant higher phonological ability. In contrast, pupils categorized as Low-math performers were particularly characterized by having a significant lower visuo-spatial ability. Findings suggest that it is important for educators to recognize and acknowledge individual differences in WM to support mathematical achievement at an individual level.

## Introduction

One topic in education that has been receiving rapidly growing attention is the learning of mathematics. In Sweden, mathematics is one of the subjects in school that has the highest failure rate among students. In a related vein, according to the National Center for Educational Statistics (National Center for Education Statistics [NCES], 2011), only 40 % of fourth-grade students and 35 % of the eight-grade students performed at or above the proficiency level (i.e., solid academic performance for the assessed grade) in math. Likewise, in international comparisons such as Trends in International Mathematics and Science Study (TIMSS, 2007, 2011), Sweden, as well as many other countries, has showed a negative trend for educational achievement in mathematics during the last decade and the Swedish government has allocated a lot of financial resources to find interventions to prevent this trend to continue. Introducing a National test in mathematics in grade three, which is the focus in the current study, is one among other political decisions.

It is widely accepted that there are individual differences in children’s cognitive ability to learn and acquire knowledge for scholastic achievement (see e.g., Engle et al., 1999; Hitch et al., 2001). Working memory (WM) is a cognitive concept thought to play a central role for the development of reading and mathematical skills. Mathematics builds on several cognitive abilities and we know from a wealth of literature that individual differences in working memory capacity (WMC) are related to mathematical performance and further academic success (Hitch et al., 2001; Reukhala, 2001; Bayliss et al., 2003; Jarvis and Gathercole, 2003; Gathercole et al., 2004; Swanson and Kim, 2007; Bull et al., 2008; Swanson et al., 2008; De Smedt et al., 2009; Gathercole and Dunning, 2010; Menon, 2010; Geary, 2011a,b; Dumontheil and Klingberg, 2012; Nyroos and Wiklund-Hörnqvist, 2012; Friso-van den Bos et al., 2013; Li and Geary, 2013; Bergman-Nutley and Klingberg, 2014), above and beyond measures of socio-economic status (Dulaney et al., 2015), language skills (Lee et al., 2004), and general intelligence (Alloway and Alloway, 2010). Despite that individual differences in WMC have been shown to influence behavioral measurements in mathematical proficiency, less is known how corresponding differences exist between the subcomponents in WM, as proposed by Baddeley (2000), and the National subtests in mathematics.

Here, we investigate individual differences in WM in relation to performance at the National curriculum tests in mathematics in a sample of Swedish third grade pupils (*N* = 597). To further investigate if levels of mathematical proficiency can be conceptualized in terms of different WM profiles, we split the sample into four groups derived from overall mathematical performance. The delineation of different WM profiles provides a more complete picture of the influence role of cognitive abilities involved in mathematics. However, it also opens up for an important empirical question: How are the different subcomponents in the original tripartite model of WM (Baddeley, 2000) related to different mathematical proficiency levels?

### Working Memory

Working memory refers to our ability to temporarily store and manipulate information needed while executing complex cognitive tasks such as numerical and arithmetic processing, problem solving and reasoning (Alloway and Alloway, 2010; Menon, 2010). The most widely used theoretical model for WM is the multicomponent model initially proposed by Baddeley and Hitch (1974) and later revised by Baddeley (2000). According to Baddeley (2000) revised model, WM is composed by four components: the central executive, the phonological loop, the visuo-spatial sketchpad and the episodic buffer (Baddeley, 2000). The central executive is a domain-general attentional control system involved in several processes such as the simultaneously storing and processing of information while handling complex tasks (e.g., mathematics). It is supported by two domain-specific slave systems: the visuo-spatial sketchpad and the phonological loop. The visuo-spatial sketchpad temporarily store visual and spatial information, whereas the phonological loop is involved in the temporarily storage and rehearsal of auditory and phonological information (Baddeley and Hitch, 1974; Baddeley, 2000). Baddeley (2000) added a fourth component, the episodic buffer. The episodic buffer is hypothesized to be responsible for integrating information from the subsystems and long term memory under the supervision of the central executive (Baddeley, 2000). Hitch et al. (2001) investigated WM through complex WM tasks and the possibility of its ability in predicting children’s school performance as measured 1 year later. They found support for the hypothesis that WMC contains a combination of domain-specific and domain-general resources, which in the Bayliss et al. (2003) study has been identified as a general processing component, verbal storage component, and a visuospatial storage component.

### Mathematics

Mathematics is one of the fundamental skills a child needs to master to successfully progress through the school years (Parsons and Bynner, 2005; Geary et al., 2012). A meta-analysis using six longitudinal datasets found that children with low mathematical ability when entering school/at school entry mostly stayed behind throughout schooling, independent of gender or socio-economic status (Duncan et al., 2007; see also Andersson, 2010; Dulaney et al., 2015 for related findings). Thus, identifying and understanding factors important for mathematical achievement will provide valuable knowledge aiding in the development of appropriate educational methods aimed at enhancing mathematical learning (Turley-Ames and Whitfield, 2003; Gersten et al., 2005; Holmes et al., 2009; Geary, 2011a; Skolverket [National Board of Education], 2011; Dulaney et al., 2015; Ekstam et al., 2015; Swanson, 2015). In a large-scale study, Bryant et al. (2000), compared how teachers rated the behavioral characteristics of 870 pupils identified as having mathematical difficulties with 854 pupils with other educational difficulties. Results showed that the difficulty in carrying out multi-step problems was one feature that specifically differentiated pupils with mathematical difficulties from the other pupils (Bryant et al., 2000), which corresponds to the function of WM.

Mathematics is an umbrella term encompassing a broad variety of competencies, tapping different rolls/functions such as switching between operations, strategies, and mental models while solving a task (Mayer, 1998; National Council of Teachers of Mathematics [NCTM], 2000; Niss, 2003; Lépine et al., 2005; Alloway, 2006; Abhakorn, 2008; Lithner, 2008; Lithner et al., 2010; Mee-yin Chan and Suk-han Ho, 2010; Lgr 11, 2011) and known to be closely related to WM (see Peng et al., 2016: for a meta analysis; Raghubar et al., 2010 for a review). Up to date, the relation between mathematics and WM has mainly been focused on WM as a potential predictor for overall mathematical performance (see Raghubar et al., 2010 for a review; Swanson and Jerman, 2006). For example Hitch et al. (2001) found that the predictability of the complex WM tasks, a year later, accounted for 27% of the variance in basic mathematical skills. However, when considering the variety of mathematical subdomains it seems likely that the contribution of WM and the different subcomponents, within Baddeley (2000) tripartite model of WM, will vary as a function of mathematical domain.

### Working Memory and Mathematics

The importance of the different subcomponents in the tripartite model of WM (Baddeley and Hitch, 1974; Baddeley, 2000) for mathematical achievement has been investigated using different designs and populations. It is widely accepted that the contribution of executive WM resources for mathematic achievement and performance are crucial, but less consistency exists about the role of the two slave systems for mathematics. Findings from longitudinal studies with young children are mixed. Using a longitudinal design, De Smedt et al. (2009) suggested that mathematical performance in first grade children are related to individual differences in visuo-spatial ability, but with a shift toward reliance on the phonological ability as a function of increased age (i.e., second grade; De Smedt et al., 2009; see also Hecht et al., 2001). In contrast, other studies have emphasized the visuo-spatial ability as crucial (Bull et al., 2008; Geary, 2011b). For example, in a sample of preschoolers (mean age: 4.6 years) Bull et al. (2008) found in their longitudinal study that visuo-spatial WM, but not phonological WM, predicted mathematical performance at the end of the third grade of primary school.

Similar findings have also been obtained in cross-sectional studies with older children (e.g., Holmes and Adams, 2006). In a sample of typically normal developing 8- and 9-year-old children, Holmes and Adams (2006) found that in both age groups, measurements of the central executive and visuo-spatial ability predicted curriculum-based mathematical performance. Moreover, for older children the measurement capturing the phonological loop predicted performance on easy mathematical tasks, but not performance on difficult tasks (Holmes and Adams, 2006). The latter finding might indicate a gradual shift toward the initial use of cognitive strategies relying on a verbal code (McKenzie et al., 2003), but the efficiency of those strategies might still be in its infancy and related to the degree of task demands (Holmes and Adams, 2006; Raghubar et al., 2010) or moderated by individual differences in WMC (Swanson, 2015).

However, some conflicting evidence exists regarding the role of the subcomponents in WM for mathematical performance related to age. Whereas some studies emphasizes the importance of verbal WM for mathematical performance with increasing age (Swanson and Kim, 2007; De Smedt et al., 2009; Van de Weijer-Bergsma et al., 2015), others found evidence for visuo-spatial WM as important (Gathercole and Pickering, 2000; Reukhala, 2001; Jarvis and Gathercole, 2003; Maybery and Do, 2003; Meyer et al., 2010). Using a cross-sectional design, Van de Weijer-Bergsma et al. (2015) found evidence for both verbal and visuo-spatial WM as equally important for mathematical performance up to grade four, but thereafter verbal WM takes over up to grade six. Thus, Meyer et al. (2010) investigated whether the contribution of the WM subcomponents changed for mathematical achievement in children at age 8- (second grade) as compared to 9-year-old children (third grade). The results showed that both the central executive and the phonological loop predicted mathematical reasoning during the second grade, but not in the third grade. Instead, the visuo-spatial component of WM was predictive for mathematical ability in third graders (Meyer et al., 2010). Correlation studies have found that the relation between the visuo-spatial WM and standardized curriculum mathematical tests persists even in older children, ranging from 7 to 14 years old (Gathercole and Pickering, 2000; Reukhala, 2001; Jarvis and Gathercole, 2003; Maybery and Do, 2003) and Reukhala (2001) found a significant correlation between mathematical performance and visuo-spatial WM even when controlling for verbal WM in a sample of adolescents 15–16 years old. Together, those results indicate the potential role of individual differences in the different subcomponents in WM for academic success across ages, but also the inconsistency findings across studies independent of study design.

The relation between the different subcomponents of WM and mathematical achievements has also been studied among children with mathematical learning difficulties (MD). Meta-analytic findings suggest that children with MD have lower verbal WM and visuo-spatial WM compared to normal achievers (Swanson and Jerman, 2006; Swanson et al., 2009). Furthermore, they found that differences in cognitive functioning between children with MD and normal achievers were primarily related to differences in verbal WM. In contrast, other studies have only found differences in visuo-spatial WM when contrasting children with MD against normal achievers (McLean and Hitch, 1999; Andersson, 2010; Andersson and Östergren, 2012), and against children with reading difficulties (Landerl et al., 2009). Based on the literature it is not clear which subcomponent of WM that is most crucial for mathematics achievement but taken together, findings indicate that individual variation in WM is associated differently depending on at least age (De Smedt et al., 2009), mathematical skills (Swanson and Jerman, 2006), and the specific mathematical domain (Träff, 2013; Kyttälä et al., 2014). It is therefore important to further investigate the different subcomponents of WM in relation to mathematical achievements (Alloway et al., 2004; see also; Bayliss et al., 2005) and in relation to different mathematical domains.

### Aim with the Current Study

The current study had two goals in mind. The first aim was to investigate the relationship between the WM subcomponents and performance in different mathematics domains (as measured by the National tests in Sweden) in a large and representative sample of Swedish grade 3 pupils. Our secondary aim was to delineate cognitive profiles in relation to mathematics performance by differentiating pupils into mathematical subgroups derived from overall mathematical performance. It was hypothesized that individual differences in WMC would predict mathematical performance overall, but less clear remained how the different subcomponents in WM were predictive for the different subtests in mathematics. Second, we hypothesized that individuals with lower mathematical performance would show a different cognitive profile compared to those performing at a higher level in mathematics. Based on prior studies and the age of the current sample, we expected children with lower mathematical proficiency to be more impaired in both visuo-spatial and phonological WM than children with higher mathematical proficiency.

## Materials and Methods

### Participants

In the present study, a total of 597 Swedish third grade pupils (*M* = 9.34 years, *SD* = 0.30) participated, 305 girls (*M* = 9.35 years, *SD* = 0.30) and 292 boys (*M* = 9.34 years, *SD* = 0.30). The sample came from 39 different regular school classes located in five different municipalities. The schools were chosen in order to represent the larger range of geographic and demographic status, based on a grouping by the Swedish Association of Local Authorities. The head teacher and teacher in, respectively, school were contacted and asked to participate. Written informed consent from parents was obtained according with the Declaration of Helsinki, and all children approved to participate. All pupils were assessed at the end of the spring term. The study was approved by the Region Ethical Review Board, Sweden.

### Materials

#### Mathematical Proficiency

Mathematical proficiency was assessed by the Swedish National tests in mathematics for grade 3 pupils. The National tests are state-mandated, curriculum-based tests given in different core subjects to pupils in grade 3, 6, and 9 in compulsory school. The National tests in mathematics for grade 3 pupils consists of seven different subtests covering several different mathematical domains to evaluate a number of syllabus goals (**Table 1**). The tasks varied in form (from plain numbers to larger tasks) as well as required different methods of expression (e.g., drawing and writing). One subtest was a group assignment and is therefore excluded in the analysis.

**Table 1:** Different syllabus goal in mathematics tested by different subtests (Skolverket [National Board of Education], 2012).

The National mathematical subtests	Syllabus goal
Algorithm and statistics	The algorithm and statistics assessment tests the pupil’s ability to use and analyze mathematical concepts, use appropriate mathematical methods and to use different mathematical forms of expression to communicate the outcome.
Fraction	The fraction assessment tests the ability to compare, rank, and divide numbers within integers 0–100 by using different types of illustrations. It also tests the ability to divide wholes into fractions, to describe, compare, identify, and naming fraction as simple fraction
Geometry	The geometry assessment tests the ability to use rudimentary geometric concepts to describe the characteristics of geometric objects, their position and compare how they relate to other geometric objects by using mathematical forms of expression to communicate.
Number understanding and Mental arithmetic	The number understanding test the pupils ability to read and write numbers and symbols, and showing digits value in numbers within integers 0–1000. It also assesses pupil’s ability to describe simple number sequences, and managing mathematical equalities within integers 0–20. The mental arithmetic assessment tests the pupil’s ability to solve mathematical problems in the four rules of arithmetic without using written working out as an aid. Numbers and answers are within integers 0–20 also some simple numbers are within an enlarged number area.
Problem solving	The assessment for problem solving tests the pupil’s ability to formulate and solve problems by using mathematical concepts and mathematical reasoning when communicate and explaining the problem as well as its outcome. The tasks require the pupil to use both symbols and words when calculating, describing and communicate the solution.
Time, area, and volume	The time, area, and volume assessment tests the ability of measuring simple comparisons, and estimations of different lengths, areas, volumes, and times, using common and appropriate units of measurement to express the results. The task tests the pupil’s ability to initiate and follow a mathematical reasoning.

As the purpose with the study was to delineate how individual differences in WMC was related to curriculum-based mathematical competencies (see **Table 1**), we analyzed tests validated and designed to assess those skills according to the National Board of Education in Sweden. The six subtests included in the analysis were: *Algorithms and Statistics* (maximum score 20, to reach solid academic performance for the assessed grade a score of 14 was required), *Fraction* (maximum score 13, to reach solid academic performance for the assessed grade a score of 8 was required), *Geometry* (maximum score 14, to reach solid academic performance for the assessed grade a score of 9 was required), *Number understanding and mental arithmetic* (maximum score 21, to reach solid academic performance for the assessed grade a score of 14 was required), *Problem solving* (maximum score 8, to reach solid academic performance for the assessed grade a score of 5 was required) and *Time, area* and *volume* (maximum score 13, to reach solid academic performance for the assessed grade a score of 8 was required). The internal consistency statistic between the subtests was good, with Cronbach’s α = 0.81 (Kline, 2000). The purpose with the National mathematical tests in Sweden are mainly summative but also intended to be used as a formative instrument in which the teacher uses the results as a pupil’s knowledge profile to further support the progress of the pupil’s mathematical proficiency at an individual level.

#### Working Memory

The measurements for WM consisted of three computerized tasks, representing both content domains of WM (verbal and spatial) and both functional aspects (storage in the context of processing and potential trade-offs between these): Operation span, Digit span, and Block span; each of which primarily evaluates one of the three components of Baddeley’s and Hitch (1974) WM model: the central executive, the phonological loop and the visuo-spatial sketchpad, respectively.

#### Operation Span

Measurements with intention to capture individuals’ WMC is often assessed by using complex span tasks, which requires participants to simultaneously process and maintain some information (Conway et al., 2005). The Unsworth et al. (2005) Operation span (Ospan) has shown good internal consistency (0.78) and test–retest reliability (0.83) and is a widely accepted measure of WMC (Turner and Engle, 1989; Klein and Fiss, 1999; Conway et al., 2005; Unsworth et al., 2005; Chein et al., 2011). In the computerized Ospan task the participants are asked to remember a series of letters while performing a concurrent task in which they judge whether a math equation is true or false (for full task descriptions, see Unsworth et al., 2005). In the current study, Ospan was age-adapted such that simpler mathematical operations were used (i.e., addition, with the sum of integers always in the range of 3–9) but with the same set of high frequency letters in the operation letter strings as in the original version of Ospan (cf. Unsworth et al., 2005). The set-sizes proceeded in fixed level from two sets with no predefined highest level, as long as two consecutive sets at any span length were correctly answered. The dependent variable was the number of correct recalled letters in the correct position (Nyroos et al., 2015).

#### Digit Span

For the measurement of the phonological WM the digit span (forward and backward) was used (McLean and Hitch, 1999). The computerized digit span was adapted from WISC-IV which has shown to have good internal consistency and re-test reliability ranging from 0.80 to 0.89 (Flanagan and Kaufman, 2004). A composite of forward and backward digit span (number of correct trials across tasks) served as our measurement for phonological WM (Baddeley, 2000; D’Amico and Guarnera, 2005). In the digit span, numbers ranging from 1 to 9 were displayed on the computer screen at a rate of one number per second. The child is asked to respond by recall the numbers in the correct order (forward and backward, respectively) by pressing the corresponding number on the keyboard. Trials increased from two to a maximum of nine numbers in length, with two trials for each span length. Testing continued until a child failed to repeat two sets at any particular span length. The raw scores for both digit span forward and backward were calculated as the number of correct trials, respectively, but collapsed into one score.

#### Block Span

For the measurement of the visuospatial WM the Block span task (or Corsi block-tapping test) was used (forward and backward). The computerized block span was adapted from WISC-IV which has shown to have good internal consistency and re-test reliability ranging from 0.80 to 0.89 (Flanagan and Kaufman, 2004). A composite of forward and backward block span (number of correct trials across tasks) served as our measurement for visuo-spatial WM (Baddeley, 2000; D’Amico and Guarnera, 2005). Block span measures an individual’s capacity to remember blocks, forward and backward, and is a commonly used measure of visuo-spatial WM (McLean and Hitch, 1999). Spatial span forward is a measure of the visual-spatial storage component of WM (i.e., the visuo-spatial sketchpad) and spatial span backward is a measure of the storage and processing components of visual-spatial WM (i.e., the visuo-spatial sketchpad plus central executive components: Lui and Tannock, 2007). In the block span, 16 green blocks were presented at the computer screen, arranged as a four-by-four square, with one block at a time randomly flashing red at a rate of one box per second. The child is then asked to remember the sequence of blocks displayed red and then respond by recall the sequence on a new square with 16 green blocks, either in the same order as presented (block span forward) or in the opposite order (block span backward). Testing continued until a child failed to repeat two sets at any particular span length, and scores were calculated in the same way as in the digit span task.

### Procedures

#### National Tests in Mathematics

The National tests in mathematics were scheduled to be administered within a 10-week period in the end of the spring term at specific dates decided by the school. The different subtests were conducted by the responsible teacher which all had received specific written instructions and scoring guidelines from The Swedish National Agency for Education (2007; in order to maintain equality in the test procedure as well as at the scoring procedure).

#### Working Memory Battery

The tasks were administered by two trained research assistants. All tasks were assessed individually in front of a computer. The tasks were administered in groups of one to three at the school. Before the session, the participants received information about the confidentiality of individual test results, verbal instructions and each task started with practice trials to ensure they understood the task. To prevent misunderstandings, the pupils were encouraged to ask questions before the assessment took place and written instructions were also provided at the computer screen before each task started during the session. The data collection for the measurements of WM was administered within a 4-month period at the end of the autumn term and ended in the beginning of the spring term just before the period when the National tests in mathematics started.

### Statistical Analysis

To investigate how the different WM subcomponents (verbal, visuo-spatial, and central executive) predicted mathematical performance across different mathematical domains, a series of multiple regression analyses were performed. To pursue to what extent mathematical proficiency differed with regard to the specific subcomponents of WM, the sample was divided into quartile groups derived from their overall mathematical performance. Instead of dividing the sample in groups based on cognitive performance, we used the total score of the National tests as cut-off criteria. This resulted in four new groups: *Low* (*n* = 144), *Low-Average* (*n* = 133), *High-Average* (*n* = 165), and *High* mathematical proficiency group (*n* = 154). The cut-off score of quartiles was motivated on the basis of using the same procedure as used in prior studies (Jordan et al., 2003; Swanson and Jerman, 2006; Cirino et al., 2015). We used a score at or below the 25th percentile as the cut-off for the group labeled as *Low*. Given the relatively large sample size in the current study, instead of collapsing children that scored at average (between the 25th and 75th percentile) into one group, we divided those into the *Low- Average* and the *High-Average* group. The *Low-Average* refer to those scoring between the 25th and 50th percentile while the *High-Average* refers to those scoring between the 50th and the 75th percentile. Finally, the fourth group, labeled as *High* scored at or above the 75th percentile. Recent research has emphasized that individual differences in cognitive ability varies a lot within the same educational grade (Van de Weijer-Bergsma et al., 2015). Here, we focused on the whole range by including a large-scale sample of children in regular schooling and within the same educational grade. Multiple group confirmatory factor analysis (CFA) was used to investigate if the WM measures were invariant (i.e., worked similarly well) across the performance groups. This is done by comparing a series of nested models from the least to the most restrictive model. If the more restrictive model does not significantly worsen the fit of the model, measurement invariance is supported. We used the chi-square (χ^2^), the comparative fit index (CFI), and the root mean square error of approximation (RMSEA) as overall model fit indices. A non-significant result for the χ^2^, values over 0.90 for CFI, and values under 0.08 for the RMSEA indicate good model fit (Marsh et al., 2004). To compare nested models, we calculated the Δχ^2^, where a non-significant result indicates that the more restrictive model fit the data as good as the comparison model.

To examine whether, and in that case how, the subcomponents in WM differed between the mathematical groups, a multivariate analysis of variance (MANOVA) was conducted for the three WM subcomponents (raw scores, see **Table 2**) with math group as between subject factor. Bonferroni correction was applied to correct for multiple testing. The rationale for this analysis was to get a better understanding of how mathematical ability can be understood in terms of WM profiles.

**Table 2:** Descriptive statistics of working memory tasks and the different National mathematical tests for the total sample and the mathematical subgroups.

	Total sample (*n* = 596)		Low Math (*n* = 144)		Low-Average (*n* = 133)		High-Average (*n* = 165)		High Math (*n* = 154)	
Measures	*M*	(*SD*)	*M*	(*SD*)	*M*	(*SD*)	*M*	(*SD*)	*M*	(*SD*)
WM (total score)	17.20	(2.99)	15.34	(2.47)	16.68	(2.96)	17.97	(2.46)	18.57	(3.00)
Operation span	3.02	(1.38)	2.50	(1.33)	2.84	(1.37)	3.27	(1.31)	3.41	(1.34)
Block span	7.51	(1.56)	6.73	(1.37)	7.36	(1.45)	7.90	(1.48)	7.96	(1.62)
Digit span	6.66	(1.28)	6.11	(1.09)	6.47	(1.33)	6.80	(1.11)	7.20	(1.33)
Mathematics (total score)	76.30	(9.41)	63.39	(9.59)	75.33	(1.30)	80.13	(1.41)	85.14	(1.76)
Algorithm and statistic	16.64	(3.03)	13.19	(2.94)	16.03	(2.10)	17.68	(1.71)	19.28	(0.98)
Fraction	11.88	(1.51)	10.42	(2.07)	11.95	(0.92)	12.27	(0.88)	12.76	(0.60)
Geometry	11.18	(1.93)	9.67	(2.18)	10.76	(1.61)	11.54	(1.46)	12.59	(1.04)
Number understanding and mental arithmetic	19.12	(2.56)	16.42	(3.54)	19.38	(1.72)	20.01	(0.99)	20.47	(0.72)
Problem solving	6.04	(1.93)	3.94	(2.13)	5.89	(1.44)	7.53	(0.74)	7.53	(0.74)
Time, area, and volume	11.44	(1.80)	9.75	(2.29)	11.32	(1.39)	12.01	(1.07)	12.51	(0.76)

*^∗^p < 0.05, ^∗∗^p < 0.01, and ^∗∗∗^p < 0.001.*

## Results

Descriptive results for the overall WM and its subcomponents as well as overall mathematical proficiency and the different mathematical domains are presented in **Table 2.** One participant was recognized as an outlier (mathematical performance below 2 SDs of the average mean) and therefore excluded from the analysis. A normal distribution analysis showed that the skewness (-1.895) and kurtosis (5.956) for the overall mathematical score was within acceptable normal distribution (Finney and Di Stefano, 2006) but with a tendency for the majority of the pupils to perform well. For the overall WM scores, the analyses of skewness (0.077) and kurtosis (0.046) revealed that the WM scores were normally distributed.

The regression analyses (see **Table 3**) were conducted using each mathematical subtest as the dependent variable and the three WM measurements as independent variables. In addition, the total mathematical score was also examined within the regression analysis.

**Table 3:** Regression analyses with mathematical subtests as dependent variables and working memory subtests as independent variables.

Dependent variables	Independent variables	*B* (*SE*)	β	*R*^2^	*F* (total model)
Math total score	Digit span	1.40 (0.30)	0.19***		
	Block span	1.41 (0.24)	0.23***		
	Operation Span	1.03 (0.26)	0.15***		
				0.17^∗∗∗^	*F*(3,593) = 40.19
Algorithms and statistics	Digit span	0.34 (0.09)	0.14**		
	Block span	0.40 (0.08)	0.20***		
	Operation span	0.23 (0.09)	0.11**		
				0.11^∗∗∗^	*F*(3,593) = 23.79
Fraction	Digit span	0.22 (0.05)	0.19***		
	Block span	0.21 (0.04)	0.22***		
	Operation span	0.08 (0.04)	0.07		
				0.13^∗∗∗^	*F*(3,593) = 28.47
Geometry	Digit span	0.13 (0.06)	0.08		
	Block span	0.11 (0.05)	0.09*		
	Operation span	0.17 (0.06)	0.12**		
				0.04^∗∗∗^	*F*(3,593) = 9.16
Number understanding and mental arithmetic	Digit span	0.33 (0.08)	0.17***		
	Block span	0.29 (0.07)	0.18***		
	Operation span	0.20 (0.08)	0.11**		
				0.11^∗∗∗^	*F*(3,593) = 23.28
Problem solving	Digit span	0.20 (0.06)	0.13**		
	Block span	0.20 (0.05)	0.16***		
	Operation span	0.24 (0.06)	0.17***		
				0.11^∗∗∗^	*F*(3,593) = 23.92
Time, area, and volume	Digit span	0.17 (0.06)	0.12**		
	Block span	0.20 (0.05)	0.18***		
	Operation span	0.12 (0.05)	0.09*		
				0.08^∗∗∗^	*F*(3,593) = 17.03

*^∗^p < 0.05, ^∗∗^p < 0.01, and ^∗∗∗^p < 0.001.*

As can be seen in **Table 3**, individual differences in WMC significantly predicted mathematical performance for all National mathematical subtests. Although, the degree of significant contribution for the different subcomponents in WM varied across mathematical subtests (see **Table 3**).

Next, to identify individual WM profiles, the sample was divided into quartile mathematical groups (*Low*, *Low-Average*, *High-Average*, and *High* mathematical group) as derived from the general mathematical proficiency score (for descriptive statistics, see **Table 2** below). For the four new math groups, there were no significant differences for gender [χ^2^(3, *N* = 596) = 1.541, *p* = 0.673] or chronological age in months [*F*(3,592) = 1.194, *p* = 0.31] between the four groups. To ensure that the WMC measurements were comparable across the four groups, we conducted multiple group CFAs. We used a model that assumed the same factor structure (one overall WM factor, and Ospan, digit span forward and backward, block span forward and backward as factor indicators) across groups but allowed the factor loadings and item intercepts to vary as the comparison model, χ^2^(20) = 12.35, *p* = 0.90; CFI = 1.00; RMSEA = 0.00. We then compared this model with a more restricted model were factor loadings were constrained to equality in all groups but item intercepts were allowed to vary, χ^2^(35) = 25.596, *p* = 0.58; CFI = 1.00; RMSEA = 0.00. Forcing the factor loadings to equality did not significantly worsen the fit of the model, Δχ^2^(15) = 13.246, *p* = 0.34. We then compared this model with a fully invariant model were both factor loadings and item intercepts were constrained to equality in all groups, χ^2^(47) = 39.008, *p* < 0.05; CFI = 1.00; RMSEA = 0.00. The fully invariant model fitted the data as well as the previous model, which clearly indicates measurement invariance across groups, Δχ^2^(12) = 13.412, *p* = 0.34. In other words, the WMC measures worked in a similar way in all four groups.

**FIGURE 1:**
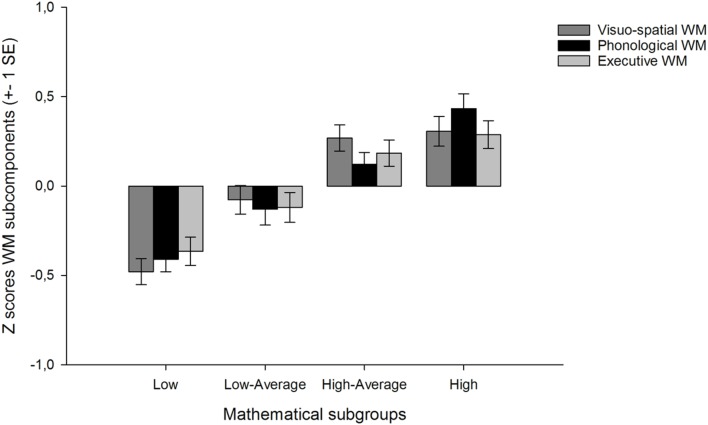
**The working memory subcomponents and mathematical subgroups.** For illustrative purpose, the figure contains the *z*-transformed working memory scores.

The MANOVA with group (*Low*, *Low-Average*, *High-Average*, and *High*) as the between subject factor and WM (visuospatial, phonological, and executive) as the dependent variables showed a significant group effect for the WM scores, Hotelling’s *T*^2^_(9,1766)_ = 14.382, *p* < 0.001. Univariate *F*-tests revealed significant group effects for all of the WM subtests. Visuospatial ability [*F*(3,592) = 22.31, *p* < 0.001, η_*p*_ = 0.102], phonological ability [*F*(3,592) = 21.74, *p* < 0.001, η_*p*_ = 0.10] and for the central executive ability [*F*(3,592) = 14.33, *p* < 0.001, η_*p*_ = 0.07]. For multiple comparisons, Bonferroni adjusted *post hoc* analyses were performed for each WM subcomponent, and Cohens *d* is reported for significant group differences. Cohens *d* of 0.2, 0.4, and 0.8 are considered as small, medium and large effect sizes, respectively (Cohen, 1992). **Figure 1** (below) depicts the *z*-transformed WM scores for each group separately.

### Visuospatial WM

For visuospatial ability, the Low mathematical group performed significantly lower at the visuospatial tasks when compared to the Low-Average group (*p* = 0.003, *d* = 0.45) and when compared to the other two groups (all *p*’s < 0.001, *d* = 0.82, respectively) indicating an overall lower visuospatial ability in those considered as poor mathematicians. The Low-Average mathematical group had significantly lower scores on the visuospatial task when compared to High-Average (*p* = 0.011, *d* = 0.37) and the High mathematical group (*p* = 0.004, *d* = 0.39) but no significant difference was found between the High-Average group and the High mathematical group (*p* > 0.05).

### Phonological WM

For phonological ability, the Low mathematical group did not differ significantly from the Low-Average group (*p* = 0.08), but performed significantly lower when compared to High-Average and the High mathematical group (all *p*’s < 0.001, *d* = 0.63 and *d* = 0.90, respectively). No significant differences between the Low-Average and High-Average (*p* = 0.129) but the High mathematical group performed significantly better at the phonological task when compared to High-Average group (*p* = 0.02, *d* = 0.33) indicating that good phonological ability seems to be important for mathematical achievement.

### WM – The Central Executive

For the complex WM task, no significant differences were found between the Low and the Low-Average group (*p* = 0.20) but the Low mathematical group showed significantly lower performance when compared to both the High-Average and the High mathematical group (all *p*’s < 0.001, *d* = 0.58 and *d* = 0. 68, respectively). The Low-Average group performed poorer when compared to both the High-Average (*p* = 0.039, *d* = 0.32) and the High mathematical group (*p* = 0.002, *d* = 0.42) but no significant differences between the High-Average and the High mathematical group.

## Discussion

The first aim with the study was to investigate the relationship between the subcomponents in WM and performance in different mathematics domains, in a sample of third grade pupils (*N* = 596) in mainstream schools. The predictive role of WM for mathematical performance, as measured by the National tests in Sweden, revealed that individual differences in WMC significantly predicted mathematical performance independent of mathematical domain. This finding confirms prior studies which has suggested a relationship between WM and National curriculum tests (Reukhala, 2001; Jarvis and Gathercole, 2003; Holmes and Adams, 2006; Nyroos and Wiklund-Hörnqvist, 2012; Friso-van den Bos et al., 2013). Our secondary aim was to explore cognitive profiles in relation to mathematics performance by differentiating pupils into mathematical subgroups derived from overall mathematical performance as measured by the National tests in Sweden. Pupils labeled as High mathematical achievers were characterized by having significantly better phonological WM, when compared to the other three groups, whereas those labeled as Low mathematical achievers were characterized by having significantly poorer visuo-spatial WM, as compared to the other three groups. These results are further elaborated below.

Regarding the outcomes from the regression analysis, all three subcomponents in WM (verbal, visuo-spatial, and central executive) significantly predicted overall mathematical performance but the significant contribution varied with respect to mathematical domain. Visuospatial WM, as measured by the Block span task, was the only subcomponent which was a significant predictor across all six mathematical domains. The present findings confirm that individual differences in visuo-spatial ability is crucial for general mathematical proficiency (Gathercole and Pickering, 2000; Reukhala, 2001; Jarvis and Gathercole, 2003; Maybery and Do, 2003; Holmes and Adams, 2006; Meyer et al., 2010; Geary, 2011b). Mathematics as a subject is rather visuo-spatial by nature in which tasks contain diagrams, geometric figures and represent quantities which commonly needs to be mentally visualized to be able to solve a math equation successfully (Li and Geary, 2013). The functional role of the visuo-spatial WM when children solves a mathematical task might be related to the use of mental representations of shapes and/or numbers involved while manipulating mathematical information which clearly put demands on visuo-spatial WM.

Hence, it is worth noting that in the current study the measurement of visuo-spatial WM included both the passive and dynamic aspect of WM (i.e., storage and processing; Meyer et al., 2010) which also might add support for prior findings of the central executive as important for mathematics (Hitch et al., 2001; Bayliss et al., 2003; D’Amico and Guarnera, 2005; Holmes et al., 2008). The predictive value of the visuo-spatial WM found in the current study might be related to the dynamic visuo-spatial WM as the task included both forward and backward block span (see Holmes et al., 2008 for related findings). However, even if there is a differentiation between passive and dynamic visuo-spatial tasks (Raghubar et al., 2010) it seems logic to combine those two when examining its relationship to curriculum-based mathematical tasks, which by nature mostly require some executive resources in terms of simultaneously maintaining and manipulating information in memory, either by the support of the phonological loop or the visuo-spatial sketchpad.

Surprisingly, the executive part of WM, as measured by a complex span task, was not a significant predictor for fraction. Again, as can be seen in **Table 3**, the strongest predictor for fraction was related to visuo-spatial WM. In line with prior research, it is plausible to suggest this outcome related to the age of the current sample. The children in the current study were all around the age of nine, and it appears as it is a differentiation approximately around this age in which older children rely more on the phonological loop and children younger than 9 years rely more heavily on the visuo-spatial sketchpad (Kyttälä et al., 2010; Van de Weijer-Bergsma et al., 2015). In that sense, chronological age (age based on the calendar) might be less important and instead favor individual differences in mental age (i.e., age based on intellectual development; Henry, 2001; Henry and MacLean, 2003; Van de Weijer-Bergsma et al., 2015).

For geometry, the only non-significant predictor was phonological WM, which probably is related to the mathematical tasks included. The majority of the tasks in geometry required the pupil to identify and simply describe the characteristics of basic geometric shapes, their position and compare how they relate to other geometric objects. Those cognitive processes might rather rely on automatic retrieval of mathematical facts from long-term memory while maintain and manipulate the visuo-spatial task-specific information in WM, and thereby more executive demanding, without specific demands on the phonological loop (Furst and Hitch, 2000; Reukhala, 2001). In sum, the results from the regression analysis confirm prior studies by emphasizing individual differences in WMC as predictive for curriculum-based National tests in mathematics. Do note that the skewness and kurtosis for the mathematical distribution indicated that most pupils performed very well, these psychometric characteristics indicate that the amount of variance explained by the WM measurements in the current study is underestimated.

An interesting result was the closer inspection of the *post hoc* classified subgroups in mathematics. When classifying children into low or high performers within the cognitive psychology research domain, this is commonly made on the basis of individuals’ performance derived from cognitive test batteries, and less commonly done on the basis of educational measurements such as the curriculum-based National tests. Therefore, to obtain an ecologically valid profile of mathematical proficiency, the results from the mandatory National curriculum tests in Sweden were used for classification.

Pupils classified as having Low mathematical proficiency performed lower on all WM tasks, but predominantly significant poorer in the task capturing visuo-spatial WM when compared to the other three groups. Those results are important in the light of prior findings from longitudinal studies (e.g., Bull et al., 2008; Geary, 2011b) which has shown that visuo-spatial ability is commonly found as predictive for mathematical ability in children at this age (Bull et al., 2008; Geary, 2011b) and lower visuo-spatial ability has been found as a feature in individuals with mathematical learning difficulties when compared to controls (McLean and Hitch, 1999; D’Amico and Guarnera, 2005; Andersson and Lyxell, 2007; Van der Ven et al., 2013). For example, D’Amico and Guarnera (2005) found evidence for impaired executive and visuo-spatial WM, but not verbal WM in 9-year-old children identified as having poor mathematical ability when compared to normally performing age-matched controls (D’Amico and Guarnera, 2005).

Thus, when delineating the cognitive profile of pupils labeled as High mathematical achievers, another cognitive profile emerged. Compared to the other three mathematical groups, small to large effect sizes between the groups were found (Low, Low-Average, and High-Average, *d* = 0.90, *d* = 0.63, and *d* = 0.33, respectively) indicating that better phonological WM was found among pupils characterized as High mathematical achievers. As indicated by some of the prior research, the relative contribution of the different subcomponents for mathematical performance changes as a function of age. Young children is assumed to rely more on the visuo-spatial component, but as they get older verbal WM gets more involved, thus recruiting the phonological loop. This have mainly been explained in terms of the amplified use of verbal strategies in which children transform numbers and symbols into a verbal code. The trade-off in ages has been suggested to arise around the age of nine, corresponding to the age in the current study.

Notably, performance on the National tests in Sweden is intended to be formative, in the sense that failure to reach set minimum criteria (according to the course syllabi) should alert educators to what mathematical domain which children have difficulties with; subsequently, receiving support in. As indicated by our results, mathematical proficiency could be conceptualized in terms of cognitive profiles indicating cognitive strengths and weakness. Identifying those are of practical significance for educational interventions and methods to further enhance learning by providing a more fine-graded picture of pupils strengths and weakness involved (Bryant et al., 2000; Witt, 2011; Ekstam et al., 2015; Swanson, 2015). Furthermore, we anticipate that this knowledge also will enable the application of appropriate strategies to support children’s learning on an individual level.

Recently, Swanson (2015) investigated the effects of an 8-week strategy intervention among third graders ability to solve problems. The results showed that strategy training had a positive impact on both problem solving and visuo-spatial WM, but the effect of strategy was moderated by individuals WMC (Swanson, 2015). Strategies containing verbal instruction in the absence of visual instruction was only beneficial for those with higher WMC (Swanson, 2015). In contrast, strategy training which contained both verbal and visual instructions produced transfer effects to a task capturing visuo-spatial WM independent of individual differences in WMC. Related to the results in the current study, strategy training might be especially beneficial for those labeled as Low mathematical achievers in the current study as they predominantly were characterized by having a lower visuo-spatial WM. In this respect, it is worth noticing that approximately 10% of pupils in the mainstream classroom are at risk of academic progress difficulties related to WM impairment (Alloway et al., 2009). Teachers play a pivotal role in providing a quality education to support children to be potential learners (Bryant et al., 2000; Gathercole and Alloway, 2008; Ekstam et al., 2015). The teacher’s knowledge and ability to identify pupils strengths and weakness is a prerequisite key to successfully progress throughout school for pupils at any level (Korhonen et al., 2014; Ekstam et al., 2015).

Taken together, our results suggest that individual differences in mathematical proficiency reflect different WM limitations and that the level of mathematical proficiency is related to qualitatively different cognitive profiles.

### Limitations and Future Challenges

Although we controlled for age related differences within the age group the study design does not warrant any conclusions from a developmental perspective. The positive kurtosis for the National test scores in mathematics pose a source for a type II error when used as the dependent variable. The positive skewness and kurtosis for the mathematical distribution, indicate that most pupils performed very well, and that the amount of variance explained by the WM measurements could be underestimated. The pupils in the current study were within the same educational level and we used curriculum based assessments for group classification. Nevertheless, the results clearly indicate that individual variations in cognitive ability is a crucial factor in determining the scholastic success level. Thus, we suggest that it is important for teachers to recognize the differing abilities pupils’ have in terms of cognitive strengths and weaknesses and subsequently tailor the learning support (level of WM load) to the individual child accordingly. Although, further studies are needed to corroborate our findings, the results from the current study suggest that the use of curriculum-based material is a potential way to enhance individual based teaching. Teaching that consider subject and domain specifics in relation to individual variability in cognition will arguable enhance educational attainments and also student’s educational engagement.

## Author Contributions

MN and BJ designed research. CW-H and JK performed research. CW-H, BJ, MN, JK, and HE analyzed the data. CW-H, BJ, MN, JK, and HE wrote the paper.

## Conflict of Interest Statement

The authors declare that the research was conducted in the absence of any commercial or financial relationships that could be construed as a potential conflict of interest.

## References

[B1] Abhakorn J. (2008). The implications of learner for second or foreign language teaching. *ARECLS* 5 186–204.

[B2] Alloway T. P. (2006). How does working memory work in the classroom? *Educ. Res. Rev.* 1 134–139.

[B3] Alloway T. P. Alloway R. G. (2010). Investigating the predictive roles of working memory and IQ in academic attainment. *J. Exp. Child Psychol.* 106 20–29. 10.1016/j.jecp.2009.11.003 20018296

[B4] Alloway T. P. Gathercole S. E. Kirkwood H. Elliott J. (2009). The cognitive and behavioural characteristics of children with low working memory. *Child Dev.* 80 606–621. 10.1111/j.1467-8624.2009.01282.x 19467014

[B5] Alloway T. P. Gathercole S. E. Willis C. S. Adams A.-M. (2004). A structural analysis of working memory and related cognitive skills in young children. *J. Exp. Child Psychol.* 87 85–170. 10.1016/j.jecp.2003.10.002 14757066

[B6] Andersson U. (2010). Skill development in different components of arithmetic and basic cognitive functions: findings from a three-year longitudinal study of children with different types of learning difficulties. *J. Educ. Psychol.* 102 115–134. 10.1037/a0016838

[B7] Andersson U. Lyxell B. (2007). Working memory deficit in children with mathematical difficulties: a general or specific deficit? *J. Exp. Child Psychol.* 96 197–228. 10.1016/j.jecp.2006.10.001 17118398

[B8] Andersson U. Östergren R. (2012). Number magnitude processing and basic cognitive functions in children with mathematical learning disabilities. *Learn. Individ. Differ.* 22 701–714. 10.1016/j.lindif.2012.05.004

[B9] Baddeley A. D. (2000). The episodic buffer: a new component of working memory? *Trends Cogn. Sci.* 4 417–423. 10.1016/S1364-6613(00)01538-2 11058819

[B10] Baddeley A. D. Hitch G. J. (1974). “Working memory,” in *The Psychology of Learning and Motivation: Advances in Research and Theory*, ed. Bower G. (New York, NY: Academic Press), 47–90.

[B11] Bayliss D. M. Jarrold C. Baddeley A. D. Leigh E. (2005). Differential constraints on the working memory and reading abilities of individuals with learning difficulties and typically developing children. *J. Exp. Child Psychol.* 92 76–99. 10.1016/j.jecp.2005.04.002 15963527

[B12] Bayliss D. M. Jarrold C. Gunn D. M. Baddeley A. D. (2003). The complexities of complex span: explaining individual differences in working memory in children and adults. *J. Exp. Psychol. Gen.* 132 71–92. 10.1037/0096-3445.132.1.71 12656298

[B13] Bergman-Nutley S. Klingberg T. (2014). Effect of working memory training on working memory, arithmetic and following instructions. *Psychol. Res.* 78 869–877. 10.1007/s00426-014-0614-0 25260390

[B14] Bryant D. P. Bryant B. R. Hammill D. D. (2000). Characteristic behaviors of students with learning disabilities who have teacher-defined math weaknesses. *J. Learn. Disabil.* 33 168–179. 10.1177/002221940003300205 15505946

[B15] Bull R. Espy K. A. Wiebe S. A. (2008). Short-term memory, working memory, and executive functioning in preschoolers: longitudinal predictors of mathematical achievement at 7 years. *Dev. Neuropsychol.* 33 205–228. 10.1080/87565640801982312 18473197 PMC2729141

[B16] Chein J. M. Moore A. B. Conway A. R. A. (2011). Domain-general mechanisms of complex working memory span. *Neuroimage* 54 550–559. 10.1016/j.neuroimage.2010.07.067 20691275

[B17] Cirino P. T. Fuchs L. S. Elias J. T. Powell S. R. Schumacher R. F. (2015). Cognitive and mathematical profiles for different forms of learning difficulties. *J. Learn. Disabil.* 48 156–175. 10.1177/0022219413494239 23851137 PMC4065636

[B18] Cohen J. (1992). A power primer. *Psychol. Bull.* 112 155–159. 10.1037/0033-2909.112.1.155 19565683

[B20] Conway A. R. A. Kane M. J. Bunting M. F. Hambrick D. Z. Wilhelm O. Engle R. W. (2005). Working memory span tasks: a methodological review and user’s guide. *Psychon. Bull. Rev.* 12 769–786. 10.3758/bf03196772 16523997

[B21] D’Amico A. Guarnera M. (2005). Exploring working memory in children with low arithmetical achievement. *Learn. Individ. Differ.* 15 189–202. 10.1016/j.lindif.2005.01.002

[B22] De Smedt B. Janssen R. Bouwens K. Verschaffel L. Boets B. Ghesquière P. (2009). Working memory and individual differences in mathematics achievement: a longitudinal study from first grade to second grade. *J. Exp. Child Psychol.* 103 186–201. 10.1016/j.jecp.2009.01.004 19281998

[B23] Dulaney A. Vasilyeva M. O’Dwyer L. (2015). Individual differences in cognitive resources and elementary school mathematics achievement: examining the roles of storage and attention. *Learn. Individ. Differ.* 37 55–63. 10.1016/j.lindif.2014.11.008

[B24] Dumontheil I. Klingberg T. (2012). Brain activity during a visuospatial working memory task predicts arithmetical performance 2 years later. *Cereb. Cortex* 22 1078–1085. 10.1093/cercor/bhr175 21768226

[B25] Duncan G. J. Dowsett C. J. Claessens A. Magnuson K. Huston A. C. Klebanov P. (2007). School readiness and later achievement. *Dev. Psychol.* 43 1428–1446. 10.1037/0012-1649.43.6.1428 18020822

[B26] Ekstam U. Linnanmäki K. Aunio P. (2015). Educational support for low-performing students in mathematics: the three-tier support model in Finnsih lower secondary schools. *Eur. J. Spec. Needs Educ.* 30 75–92. 10.1080/08856257.2014.964578

[B27] Engle R. W. Kane M. J. Tuholski S. W. (1999). “Individual differences in working memory capacity and what they tell us about controlled attention, general fluid intelligence, and functions of the prefrontal cortex,” in *Models of Working Memory*, eds Miyake1 A. Shah P. (Cambridge: Cambridge University Press).

[B28] Finney S. J. Di Stefano C. (2006). “Non-normal and categorical data in structural equation modeling,” in *Structural Equation Modeling: A Second Course*, eds Hancock G. R. Mueller R. O. (Charlotte, NC: Information Age Publising), 269–314.

[B29] Flanagan D. P. Kaufman A. S. (2004). *Essentials of WISC-IV Assessment.* Hoboken, NJ: John Wiley & Sons.

[B30] Friso-van den Bos I. Van der Ven S. H. G. Kroesbergen E. H. Van Luit J. E. H. (2013). Working memory and mathematics in primary school children: a meta-analysis. *Educ. Res. Rev.* 10 29–44. 10.1016/j.edurev.2013.05.003

[B31] Furst A. Hitch G. J. (2000). Separate roles for executive and phonological components in mental arithmetic. *Mem. Cogn.* 28 774–782. 10.3758/BF03198412 10983451

[B32] Gathercole S. E. Alloway T. P. (2008). *Working Memory and Learning. A Practical Guide for Teachers.* London: Sage Press.

[B33] Gathercole S. E. Dunning D. L. (2010). *Developmental Disorders and Interventions. Advances in Child Development and Behavior*, Vol. 39 Amserdam: Elsevier

[B34] Gathercole S. E. Pickering S. J. (2000). Working memory deficits in children with low achievements in the national curriculum at 7 years of age. *Br. J. Educ. Psychol.* 70 177–194. 10.1348/000709900158047 PMID: 10900777 10900777

[B35] Gathercole S. E. Pickering S. J. Knight C. Stegmann Z. (2004). Working memory skills and educational attainment: evidence from national curriculum assessments at 7 and 14 years of age. *Appl. Cogn. Psychol.* 18 1–16. 10.1002/acp.934

[B36] Geary D. C. (2011a). Consequences, characteristics, and causes of mathematical learning disabilities and persistent low achievement in mathematics. *J. Dev. Behav. Pediatr.* 32 250–263. 10.1097/DBP.0b013e318209edef PMID: 21285895 21285895 PMC3131082

[B37] Geary D. C. (2011b). Cognitive predictors of achievement growth in mathematics: a five year longitudinal study. *Dev. Psychol.* 47 1539–1552. 10.1037/a0025510 21942667 PMC3210883

[B38] Geary D. C. Hoard M. K. Nugent L. Bailey D. H. (2012). Mathematical cognition deficits in children with learning disabilities and persistent low achievement: a five-year prospective study. *J. Educ. Psychol.* 104 206–223. 10.1037/a0025398 27158154 PMC4855881

[B39] Gersten R. Jordan N. C. Flojo J. R. (2005). Early identification and interventions for students with mathematics difficulties. *J. Learn. Disabil.* 38 293–304. 10.1177/00222194050380040301 16122059

[B40] Hecht S. A. Torgesen J. K. Wagner R. K. Rashotte C. A. (2001). The relations between phonological processing abilities and emerging individual differences in mathematical computation skills: a longitudinal study from second to fifth grades. *J. Exp. Child Psychol.* 79 192–227. 10.1006/jecp.2000.2586 11343408

[B41] Henry L. MacLean M. (2003). Relationships between working memory, expressive vocabulary and arithmetical reasoning in children with and without intellectual disabilities. *Educ. Child Psychol.* 20 51–63.

[B42] Henry L. A. (2001). How does the severity of a learning disability affect working memory performance? *Memory* 2001 233–247. 10.1080/09658210042000085 PMID: 11594349 11747580

[B43] Hitch G. J. Towse J. N. Hutton U. (2001). What limits children’s working memory span? Theoretical accounts and applications for scholastic development. *J. Exp. Psychol. Gen.* 130 184–198. 10.1037/0096-3445.130.2.184 11409098

[B44] Holmes J. Adams J. W. (2006). Working memory and children’s mathematical skills: implications for mathematical development and mathematics curricula. *Educ. Psychol.* 26 339–366. 10.1080/01443410500341056

[B45] Holmes J. Adams J. W. Hamilton C. J. (2008). The relationship between visuospatial sketchpad capacity and children’s mathematical skills. *Eur. J. Cogn. Psychol.* 20 272–289. 10.1080/09541440701612702

[B46] Holmes J. Gathercole S. E. Dunning D. L. (2009). Adaptive training leads to sustained enhancement of poor working memory in children. *Dev. Sci.* 12 9–15. 10.1111/j.1467-7687.2009.00848.x 19635074

[B47] Jarvis H. L. Gathercole S. E. (2003). Verbal and nonverbal working memory and achievements on national curriculum tests at 11 and 14 years of age. *Educ. Child Psychol.* 20 123–140.

[B48] Jordan N. C. Hanich L. B. Kaplan D. Klein K. Fiss W. (2003). Arithemtic fact mastery in young children: a longitudinal investigation. *J. Exp. Child Psychol.* 85 103–119. 10.1016/S0022-0965(03)00032-8 12799164 PMC2791329

[B49] Klein K. Fiss W. (1999). The reliability and stability of the Turner and Engle working memory task. *Behav. Res. Methods Instrum. Comput.* 31 429–432. 10502865 10.3758/bf03200722

[B50] Kline P. (2000). *The Handbook of Psychology Testing*, 2nd Edn London: Routledge.

[B51] Korhonen J. Linnanmäki K. Aunio P. (2014). Learning difficulties, academic wellbeing and educational dropout: a person-centred approach. *Learn. Individ. Differ.* 31 1–10. 10.1016/j.lindif.2013.12.011

[B52] Kyttälä M. Aunio P. Hautamäki J. (2010). Working memory resources in young children with mathematical difficulties. *Scand. J. Psychol.* 51 1–15. 10.1111/j.1467-9450.2009.00736.x 19674399

[B53] Kyttälä M. Aunio P. Lepola J. Hautamäki J. (2014). The role of the working memory and language skills in the prediction of word problem solving in 4- to 7-year-old children. *Educ. Psychol.* 34 674–696. 10.1080/01443410.2013.814192

[B54] Landerl K. Fussenegger B. Moll K. Willburger E. (2009). Dyslexia and dyscalculia: two learning disorders with different cognitive profiles. *J. Exp. Psychol.* 103 309–324. 10.1016/j.jecp.2009.03.006 19398112

[B55] Lee K. Ng S.-F. Ng E.-L. Lim Z.-Y. (2004). Working memory and literacy as predictors of performance on algebraic word problems. *J. Exp. Child Psychol.* 89 140–158. 10.1016/j.jecp.2004.07.001 15388303

[B56] Lépine R. Bernardin S. Barrouillet P. (2005). Attention switching and working memory spans. *Eur. J. Cogn. Psychol.* 17 329–345. 10.1080/09541440440000014

[B57] Lgr 11 (2011). *Läroplan för Grundskolan, Förskoleklassen Och Fritidshemmet.* Stockholm: Skolverket.

[B58] Li Y. Geary D. C. (2013). Developmental gains in visuospatial memory predict gains in mathematics achievement. *PLoS ONE* 8:e70160 10.1371/journal.pone.0070160 PMC3729464 23936154

[B59] Lithner J. (2008). A research framework for creative and imitative reasoning. *Educ. Stud. Math.* 67 255–276. 10.1007/sl0649-007-9104-2

[B60] Lithner J. Bergqvist E. Bergqvist T. Boesen J. Palm T. Palmberg B. (2010). “Mathematical Competencies – a research framework,” in *Proceedings of MADIF 7 the Seventh Mathematics Education Research Seminar*, *Mathematics and Mathematics Education: Cultural and Social Dimensions*, eds Bergsten, Jablonka, and Wedege (Linköping: SMDF), 157–167.

[B61] Lui M. Tannock R. (2007). Working memory and inattentive behavior in a community sample of children. *Behav. Brain Funct.* 3 1–11. 10.1186/1744-9081-3-12 17319951 PMC1820786

[B62] Marsh H. Hau K. T. Wen Z. (2004). In search for golden rules: comment on the hypothesis testing approaches to setting cutoff values for fit indexes and dangers in overgeneralising Hu & Bentler’s (1999) findings. *Struct. Equ. Model.* 11 320–341. 10.1207/s15328007sem1103_2

[B63] Maybery M. T. Do N. (2003). Relationships between facets of working memory and performance on a curriculum-based mathematics test in children. *Educ. Child Psychol.* 20 77–92.

[B64] Mayer R. (1998). Cognitive, metacognitive, and motivational aspects of problem solving. *Cogn. Sci.* 26 49–63. 10.1348/2044-8279.002008

[B65] McKenzie B. Bull R. Gray C. (2003). The effects of phonological and visual–spatial interference on children’s arithmetical performance. *Educ. Child Psychol.* 20 93–108.

[B66] McLean J. F. Hitch G. J. (1999). Working memory impairments in children with specific arithmetic learning difficulties. *J. Exp. Psychol.* 74 240–260. 10.1006/jecp.1999.2516 10527556

[B67] Mee-yin Chan B. Suk-han Ho C. (2010). The cognitive profile of Chinese children with mathematical difficulties. *J. Exp. Child Psychol.* 107 260–279. 10.1016/j.jecp.2010.04.016 20580379

[B68] Menon V. (2010). Developmental cognitive neuroscience of arithmetic: implications for learning and education. *ZDM* 42 515–525. 10.1007/s11858-010-0242-0 22003371 PMC3193278

[B69] Meyer M. L. Salimpoor V. N. Wu S. S. Geary D. C. Menon V. (2010). Differential contribution of specific working memory components to mathematics achievement in 2nd and 3rd graders. *Learn. Individ. Differ.* 20 101–109. 10.1016/j.lindif.2009.08.004 21660238 PMC3109434

[B70] National Center for Education Statistics [NCES] (2011). *The Nation’s Report Card: Mathematics 2011(NCES 2012–458).* Washington, D.C: Institute of Education Sciences, U.S. Department of Education.

[B71] NCTM [National Council of Teachers of Mathematics] (2000). *Principles and Standards for School Mathematics.* Reston, VA: NCTM.

[B72] Niss M. (2003). “Mathematical competencies and the learning of mathematics: the Danish: KOM project,” in *Proceedings of the Third Mediterranean Conference on Mathematics Education*, eds Gagatsis A. Papastavridis S. (Athens: Hellenic Mathematical Society and Cyprus Mathematical Society), 115–124.

[B73] Nyroos M. Jonsson B. Korhonen J. Eklöf H. (2015). Children’s mathematical achievement and its relation to working memory, test anxiety, self-regulation: a person-centered approach. *Educ. Inq.* 6 73–97. 10.3402/edui.v6.26026

[B74] Nyroos M. Wiklund-Hörnqvist C. (2012). The association between working memory and educational attainment as measured in different mathematical subtopics in the Swedish national assessment: primary education. *Educ. Psychol. Int. J. Exp. Educ. Psychol.* 32 239–256. 10.1080/01443410.2011.643578

[B75] Parsons S. Bynner J. (2005). *Does Numeracy Matter More?* London: National Research and Development Centre for Adult Literacy and Numeracy.

[B76] Peng P. Namkung J. Barnes M. Sun C. (2016). A meta-analysis of mathematics and working memory: moderating effects of working memory domain, type of mathematics skill, and sample characteristics. *J. Educ. Psychol. Adv.* 108 455–473. 10.1037/edu0000079

[B77] Raghubar K. P. Barnes M. A. Hecht S. A. (2010). Working memory and mathematics: a review of developmental, individual difference, and cognitive approaches. *Learn. Individ. Differ.* 20 110–122. 10.1016/j.lindif.2009.10.005

[B78] Reukhala M. (2001). Mathematical skills in ninth-graders: relationship with visuospatial abilities and working memory. *Educ. Psychol.* 21 387–399. 10.1080/01443410120090786

[B79] Skolverket [National Board of Education] (2011). *Curriculum for the Compulsory School, Preschool Class and Recreation Centre.* Available at: www.skolverket.se/publikationer

[B80] Skolverket [National Board of Education] (2012). *Lärarinformation: Ämnesprov, Matematik, Årskurs 3 Vårtermin 2012 [Teacher Information: Subject Test, Mathematics, Grade 3 Spring term 2012].* Stockholm: Skolverket.

[B81] Swanson H. L. (2015). Cognitive strategy interventions improve word problem solving and working memory in children with math disabilities. *Front. Psychol.* 6:1099 10.3389/fpsyg.2015.01099 PMC4523823 26300803

[B82] Swanson H. L. Jerman O. (2006). Math disabilities: a selective meta-analysis of the literature. *Rev. Educ. Res.* 76 249–274. 10.1016/j.ridd.2015.01.002

[B83] Swanson H. L. Jerman O. Zheng X. (2008). Growth in working memory and mathematical problem solving in children at risk and not at risk for serious math difficulties. *J. Educ. Psychol.* 100 343–379. 10.1037/0022-0663.100.2.343

[B84] Swanson H. L. Jerman O. Zheng X. (2009). Math disabilities and reading disabilities: can they be separated? *J. Psychoeduc. Assess.* 27 175–196. 10.1177/0734282908330578

[B85] Swanson L. Kim K. (2007). Working memory, short-term, and naming speed as predictors of children’s mathematical performance. *Intelligence* 35 151–168. 10.1016/j.intell.2006.07.001

[B86] The Swedish National Agency for Education (2007). *Trends in (International) Mathematics and Science Study (TIMSS) Report 323.* Stockholm: Skolverket.

[B87] TIMSS (2007). *Assessment. Copyright* (2009) International Association for the Evaluation of Educational Achievement (IEA). Chestnut Hill, MA: TIMSS & PIRLS International Study Center.

[B88] TIMSS (2011). *Assessment. Copyright* (2013) International Association for the Evaluation of Educational Achievement (IEA). Chestnut Hill, MA: TIMSS & PIRLS International Study Center.

[B89] Träff U. (2013). The contribution of general cognitive abilities and number abilities to different aspects of mathematics in children. *J. Exp. Child Psychol.* 116 139–156. 10.1016/j.jecp.2013.04.007 23773916

[B90] Turley-Ames K. J. Whitfield M. M. (2003). Strategy training and working memory task performance. *J. Mem. Lang.* 49 446–468. 10.1016/S0749-596X(03)00095-0

[B91] Turner M. L. Engle R. W. (1989). Is working memory capacity task dependent? *J. Mem. Lang.* 28 127–154. 10.1016/0749-596X(89)90040-5

[B92] Unsworth N. Heitz R. P. Schrock J. C. Engle R. W. (2005). An automated version of the operation span task. *Behav. Res. Methods* 37 498–505. 10.3758/BF03192720 16405146

[B93] Van de Weijer-Bergsma E. Kroesbergen E. H. Van Luit J. E. H. (2015). Verbal and visual-spatial working memory and mathematical ability in different domains throughout primary school. *Mem. Cogn.* 43 367–378. 10.3758/s13421-014-0480-4 PMC4555215 25377509

[B94] Van der Ven S. H. G. Van der Maas H. L. J. Straatemeier M. Jansen B. R. J. (2013). Visuospatial working memory and mathematical ability at different ages throughout primary school. *Learn. Individ. Differ.* 27 182–192. 10.1016/j.lindif.2013.09.003

[B95] Witt M. (2011). School based working memory training: preliminary finding of improvement in children’s mathematical performance. *Adv. Cogn. Psychol.* 7 7–15. 10.2478/v10053-008-0083-3 21818243 PMC3149917

